# The Roles of Shame and Guilt in the Development of Aggression in Adolescents With and Without Hearing Loss

**DOI:** 10.1007/s10802-021-00769-1

**Published:** 2021-02-24

**Authors:** Evelien Broekhof, Marieke G. N. Bos, Carolien Rieffe

**Affiliations:** 1 Developmental Psychology, Leiden University, Leiden, The Netherlands; 2 Department of Human Media Interaction, Faculty of Electrical Engineering, Mathematics and Computer Science, University of Twente, Enschede, The Netherlands; 3 Department of Psychology and Human Development, Institute of Education, University College London, London, UK

## Abstract

This longitudinal study examined how shame and guilt contribute to the development of reactive and proactive aggression in adolescents with and without hearing loss. Adolescents between 9 and 16 years old (adolescents with hearing loss (*n* = 80; *M*age = 11.91) and without hearing loss (*n* = 227; *M*age = 11.63)) completed self-reports on three occasions with an interval of 9 months. Mixed model analyses revealed that both reactive aggression and proactive aggression decreased with age, whereas shame and guilt peaked in early adolescence. Adolescents with hearing loss reported higher levels of proactive aggression, lower levels of shame and guilt, and showed protracted development for guilt compared to their hearing peers. In both groups, shame contributed to an increase in reactive aggression, whereas guilt contributed to a decrease in proactive aggression. These longitudinal associations highlight the unique role that shame and guilt play in the development of adolescent aggression.

Aggression is any form of behaviour that has the goal of harming or injuring someone else (Bushman & Anderson, 2001). The role of shame and guilt has often been emphasized in the etiology of aggression (Malti & Krettenauer, 2013). These negative social emotions can be thought of as “gate keepers” for a better society (De Waal, 2009). For example, anticipation of the negative feeling of guilt is usually enough to prompt an individual to think twice before harming someone else. In other words, these emotions tend to make us behave within the limits set by society, and as “good citizens” who respect other peoples’ integrity and possessions. However, the longitudinal relation between the development of shame and guilt in the development of aggression still needs to be examined.

A contributing factor to the development of shame and guilt and aggression could be one’s degree of access to the social world. Adolescents with hearing loss face a unique developmental situation, providing an opportunity to examine the role of social access. Most adolescents with hearing loss grow up in a predominantly hearing world, with hearing families (Mitchell & Karchmer, 2004). Communication is generally less frequent and of a lower quality between children with hearing loss and their hearing family members or care-givers (Ambrose et al., 2015). These adolescents therefore have fewer opportunities to engage in either explicit or incidental learning, due to the limits their hearing loss imposes on overhearing others in noisy environments, on language skill development, and on the overall level of communication (Lederberg et al., 2013; Tomblin et al., 2015). Consequently, these communication difficulties are assumed to affect the social-emotional adjustment of these children.

Shame and guilt are learned within a social environment through observation, modelling, and verbal transmission (Eisenberg, 2000). Therefore, the development of shame and guilt could prove challenging for those with limited social access, as is the case for adolescents with hearing loss (Eisenberg, 2007; McCreery et al., 2015). In the present study, we compared adolescents with and without hearing loss, and we used group differences as a proxy to examine the role of social access in the development of aggression. The aims of the present study were to examine and compare (1) the levels and development of aggression and shame and guilt in adolescents with and without hearing loss, and (2) the extent to which shame and guilt contribute to the development of aggression in each group.

## Aggression

Longitudinal studies mapping the developmental course of aggression have shown that engagement in aggression starts to emerge before children reach the age of two, and this behaviour reaches a peak between the age of two and four. From this peak on, aggression starts to gradually decrease as children learn to better regulate their behaviours (Campbell et al., 2006; Côté et al., 2006; Tremblay, 2000). This decrease in aggression continues throughout childhood and adolescence (Barker et al., 2006; Bongers et al., 2004; Vierikko et al., 2006).

Research on aggression differentiates between reactive aggression and proactive aggression, due to underlying differences in motives (Cima et al., 2013; Kempes et al., 2005). Reactive aggression is a defensive response to perceived provocation or threat. This hot-tempered, impulsive type of aggression is accompanied by negative affective states, such as frustration and anger (Dodge, 1991). In contrast, proactive aggression is goal-oriented, and motivated by the desire to obtain a desired outcome (Bandura, 1973; Dollard et al., 1939). It occurs in the absence of provocation and emotional arousal.

Previous studies generated support for a differential link between reactive and proactive aggression, respectively, and children’s social information processing (SIP model: Arsenio et al., 2009). That is, a bias in interpreting social cues predicts the development of reactive aggression, but not proactive aggression. In particular, misinterpreting others’ intentions as hostile in ambiguous or benign social situations relates to higher levels of reactive aggression (Orobio de Castro et al., 2002; Dodge & Coie, 1987). In contrast, proactive aggression is linked to biases toward instrumental over interpersonal goals, and to positive expectations about obtaining instrumental goals by means of aggression (Hubbard et al., 2001).

A higher incidence of aggression has been reported in adolescents with hearing loss (e.g., Van Eldik, 2005; Chao et al., 2015).Yet these studies did not differentiate between reactive and proactive aggression. Adolescents with hearing loss may be at higher risk for developing reactive aggression, because they attribute twice as many hostile intentions to story characters in ambiguous and benign social situations as their hearing peers (Torres et al., 2016). Furthermore, adolescents with hearing loss also seem to infer that relationships are not necessarily harmed by anger or aggression (Rieffe & Meerum Terwogt, 2006; Torres et al., 2016). In contrast to hearing peers, adolescents with hearing loss did not think their friendships would be jeopardized if they were to express their anger in a peer conflict situation (Rieffe & Meerum Terwogt, 2006). In a study by Torres and colleagues (2016), adolescents were shown videos in which a protagonist acted aggressively towards a peer. Adolescents with hearing loss thought that their peers would be less inclined to reject them if they were to display aggressive behaviour compared to their hearing peers. To our knowledge, no studies have examined whether adolescents with hearing loss are at a higher risk for developing proactive aggression. But the above described findings suggest that adolescents with hearing loss might view aggressive behavior as a more attractive behavioral option to obtain instrumental goals compared to their hearing peers, since they do not attach the same level of negative repercussions to anger and aggression.

## The Development of Shame and Guilt

Shame and guilt are negative emotions typically arising following moral transgressions. Shame focusses on the fear of being negatively evaluated by others, whereas guilt focusses on the responsibility for the harm caused to another (Olthof, 2012; Tracy & Robins, 2004). Children are not born with the ability to experience these emotions. The first basic experience of shame and guilt typically occur around the age of three. The onset and development of shame and guilt depends on the acquisition of several cognitive skills: (1) a sense of self-awareness, and the capacity to reflect on the self, (2) knowledge about social rules and the capacity to evaluate one’s own behaviour according to these standards, and (3) perspective taking abilities (Muris & Meesters, 2014; Tracy & Robins, 2007).

A basic sense of self-awareness develops around two years of age (Thompson, 2006), and children rapidly learn about social rules from the age of one. Learning social rules is highly depended on input from the social environment, because children learn social rules by observing how others evaluate their behaviour or by observing how others’ behaviours are evaluated (Lagattuta & Thompson, 2007; Thompson et al., 2006). Positive behaviours are typically positively reinforced by others in the child’s social environment whereas negative behaviours will be discouraged. By means of these interactions, children will develop an understanding of the social rules (Fivush & Nelson, 2006). To experience shame and guilt, one must also be able to understand others’ perspectives and feeling states since, shame and guilt are typically experienced when one evaluates how others evaluate the self. Around the age of four, children have developed a basic understanding of others’ intentions, desires and beliefs (Peterson et al., 2005).

The acquisition of the cognitive skills for the experience of shame and guilt is reliant on input from the social world. If a child displays behaviour violating the social rules, parents typically use imperative language, a negative affective tone or will explain that displayed behaviour is unwanted (Ketelaar et al., 2015). Children with hearing loss face difficulties in these interactions, as parents are often not proficient in sign language (Mitchell & Karchmer, 2004). In addition, it is more difficult for children with hearing loss to overhear interactions to learn how others’ behaviours are evaluated. Children with hearing loss are therefore assumed to have less awareness of social rules and standards (Ketelaar et al., 2015). Children with hearing loss are also known for difficulties in perspective taking. The development of perspective taking abilities is also stimulated by verbal interactions (de Villiers & de Villiers, 2014). For example, parents name others’ mental states (*he likes swimming)*, explain behaviour based on mental states, and actively stimulate children’s perspective taking abilities (*how would you feel, if he did that to you?)*. Thus, communication about the social world around the child is crucial to the development of shame and guilt. But many children with hearing loss cannot access this kind of full communication. Not surprisingly, cross-sectional studies have indicated lower levels of shame and guilt in children with hearing loss (Ketelaar et al., 2015). Since difficulties in perspective taking abilities persist into adolescence (Gonzalez et al., 2007; Ketelaar et al., 2015), it is also important to examine the development of shame and guilt in adolescents with hearing loss.

## The Link Between Shame, Guilt and Aggression

Whether children and adolescents anticipate positive emotions (e.g., happiness) or negative emotions (e.g., shame or guilt) following imagined moral transgressions is an important predictor of aggression (Arsenio et al., 2012; Krettenauer & Eichler, 2006). The expectation that they themselves or someone else will experience positive emotions following a moral transgression is associated with higher levels of aggression, while the expectation that one will experience negative emotions following a moral transgression turns aggression into a less desirable behavioural alternative (for a meta-analysis see Malti & Krettenauer, 2013). The happy victimizer phenomenon occupies a well-known childhood phase in the development of emotion attributions. Although children around the age of four acknowledge that moral transgressions are wrong, they nevertheless attribute solely positive feelings to themselves in hypothetical situations or to hypothetical story characters (Arsenio et al., 2006). In middle childhood, children start to anticipate shame and guilt, due to an increased focus on others’ emotions and perspectives (Sokol & Chandler, 2003). However, longitudinal studies indicate that emotion attributions following moral transgressions are still developing during adolescence. Negative emotion attributions become more frequent throughout adolescence and early adulthood (Krettenauer et al., 2014; Nunner-Winkler, 2009).

Results of cross-sectional studies examining the link between aggression and shame in adolescents have been inconsistent. Some studies have indicated that shame is an unpleasant emotion, and mere anticipation of shame prevents aggressive behaviours (Olthof, 2012; Roos et al., 2011). However, others have found that shame is related to higher levels of aggression (Stuewig et al., 2010). Yet the distinction between reactive and proactive aggression could explain these inconsistent findings regarding shame. Given that ashamed individuals feel judged, and are worried about damage to their image in front of others, they may react with hostility and aggression toward disapproving others, as a means of protecting self-esteem and reinforcing a sense of superiority (Thomaes et al., 2011). This would hint at an increase in reactive aggression. However, in the absence of feeling ‘attacked’ by others, shame could evoke a feeling of having harmed one or more others, thus contributing to a decrease of proactive aggression (Olthof, 2012).

Guilt in response to wrongdoing is consistently associated with lower levels of aggression in cross-sectional studies (e.g., Stuewig et al., 2010). Guilt reflects the anticipation that one’s actions have negative consequences for others. This consideration, combined with the anticipated unpleasantness of guilt, makes it less likely that adolescents will behave immorally or aggressively (Malti, 2016). Moreover, this consequential analysis is more likely to occur in unprovoked situations. Therefore, higher levels of guilt are linked to lower levels of proactive aggression specifically (Chaux et al., 2012; Frick et al., 2003).

To examine whether the development of shame and guilt attribution co-occurs with the development of reactive and proactive aggression, longitudinal studies are needed. Previous longitudinal studies have already established that a broad range of social-cognitive, behavioural and environmental factors influence the development of reactive and proactive aggression, such as sensation seeking, popularity, lack of parental monitoring and emotional dysregulation (Brendgen et al., 2006; Cui et al., 2016; Skripkauskaite et al., 2015; Stoltz et al., 2016). However, longitudinal studies examining a possible role for shame and guilt in the development of aggression in adolescence are scarce. One study by Roos and colleagues (2014) assessed self-reported shame- and guilt-proneness and peer-nominated aggression at two time points, with a six-month interval. Although shame and guilt were both related to lower levels of aggression at the first measurement occasion, these emotions did not forecast changes in aggression over time (Roos et al., 2014). In examining the relation of shame and guilt with aggression, no longitudinal studies have differentiated between reactive and proactive aggression.

## The Present Study

In this longitudinal study, adolescents between 9 and 16 years old, with and without hearing loss, completed self-report questionnaires on three measurement occasions. An advantage of including both adolescents with and without hearing loss was that we could examine the role of shame and guilt alongside the role of social access (i.e., through group comparisons) in the development of reactive and proactive aggression. Adolescents with hearing loss have less access to the social world solely due to limitations on auditory input. It is therefore plausible that differences between both groups are the result of less access to the social world. Group differences in this study are therefore viewed as a proxy for social access.

The first aim of this study was to compare the levels and development of proactive and reactive aggression and, shame and guilt between adolescents with and without hearing loss. We expected higher levels of reactive and proactive aggression, and lower levels of shame and guilt in adolescents with hearing loss compared to their hearing peers (Ketelaar et al., 2015; Chao et al., 2015). For both groups, we hypothesised decreases in the level of reactive and proactive aggression (Barker et al., 2006; Bongers et al., 2004; Vierikko et al., 2006). In addition, we expected shame and guilt to increase throughout adolescence (Krettenauer et al., 2014), but at a slower pace in adolescents with hearing loss, as compared to hearing adolescents.

The second aim of this study was to examine the extent to which shame and guilt contributed to the prediction of reactive and proactive aggression in adolescents with and without hearing loss. Based on previous cross-sectional studies, we expected shame to contribute to an increase in reactive aggression (Thomaes et al., 2011), and both shame and guilt to contribute to a decrease in proactive aggression (Chaux et al., 2012; Frick et al., 2003). Finally, we expected these relations to be similar in adolescents with hearing loss and without hearing loss.

## Method

### Participants

80 adolescents with hearing loss and 227 adolescents without hearing loss participated in this study (see Table 1 for participant characteristics). The data presented here are part of a longitudinal study on the social-emotional development of adolescents with hearing loss. Cross-sectional studies were previously presented by e.g., Broekhof and colleagues (2020) and Theunissen and colleagues (2015). Detailed information on the population with hearing loss that is studied longitudinally can be found in Broekhof and colleagues (2018).

**Table 1:** Demographic characteristics of participants

	HL	Hearing
No. of participants	80	227
Age in years at T1		
Mean (*SD*)	11.91 (1.62)	11.63 (1.38)
Range	9.17–15.75	9.08–14.75
Gender – *n* (%)		
Male	37 (46.25)	96 (42.29)
Female	43 (53.75)	131 (57.71)
IQ score (*SD*)	10.19 (2.67)	10.61 (2.48)
Language score (*SD*)	10.29 (3.30)	10.32 (2.30)
Parental education level* (*SD*)	3.21 (.72)	3.17 (.66)
Type of education – *n* (%)		
Mainstream education	48 (60)	227 (100)
Special education	32 (40)	0
Communication mode – *n* (%)		
Dutch Sign Language /Sign Supported Dutch	28 (35)	
Spoken Language only	52 (65)	
Type of amplification—*n* (%)		
Hearing aid	53 (66.25)	
Cochlear implant (CI)	27 (33.75)	
Hearing loss in best ear – *n* (%)		
40–60 dB	20 (25.00)	
61–90 dB	18 (22.50)	
> 90 dB	36 (45.00)	
Unknown	6 (7.50)	

*The highest level of education of each parent was categorized on a scale ranging from one to four. Social economic status was calculated by averaging these two scores

*HL* Hearing loss, *SD* Standard Deviation, *T *Time

Adolescents with hearing loss were recruited via ENT departments of hospitals, special needs schools, speech and hearing centres, and magazines or websites. Since adolescents with hearing loss will be used as a proxy for social access, inclusion criteria for adolescents with hearing loss were: 1) an unaided hearing loss of at least 40 dB in the better ear. An individual with a loss of 40 dB has difficulties hearing normal speech even at close distances. 2) The hearing loss had to be detected before the age of four, meaning that the hearing loss occurred before or during language acquisition. 3) All adolescents were born to hearing parents not proficient in sign language. Adolescents without hearing loss were recruited from primary and secondary schools in the Netherlands. Inclusion criteria for both adolescents with and without hearing loss were 1) age between 9 and 16 years at Time 1 (T1), 2) average intellectual functioning, 3) no diagnosed developmental disabilities or learning difficulties, and 4) living in the Netherlands or the Dutch speaking part of Belgium. Parents indicated on the informed consent form whether their child was currently or previously diagnosed with any psychiatric condition or learning difficulty. Intellectual functioning was assessed by using two nonverbal subtests of the Wechsler Intelligence Scale for Children – Third Edition (WISC III; Wechsler, 1991): block design and picture arrangement. In addition, teachers confirmed that all participating children show intellectual functioning within the average range or above.

Hearing adolescents were all recruited from mainstream schools, and DHH adolescents were recruited from both mainstream and special education. 48 adolescents with hearing loss attended mainstream education and 32 adolescents with hearing loss attended special education (see Table 1). Special schools for children with hearing loss in the Netherlands teach both in spoken language supported by sign and in sign language. Adolescents with and without hearing loss did not differ in terms of terms of age at T1, gender distribution, IQ, language, or parental education level (see Table 1).

## Materials

*Wechsler Intelligence Scale for Children – Third edition (WISC III;* Wechsler, 1991). The block design and picture arrangement subtests were used to assess and confirm average intellectual functioning. In the block design subtest, children were asked to replicate a displayed geometrical pattern by rearranging white and red sided cubes. In the picture arrangement subtest, children were requested to sequence cartoon pictures in order to create a story in a chronological order. The obtained scores for the subtests were converted into age-corrected norm scores and the grand population mean was set to 10. An IQ score was calculated for each child based on these two norm scores (see Table 1).

*Instrument for Reactive and Proactive Aggression (IRPA) Self-Report* (Rieffe et al., 2016): Adolescents were asked to report their aggressive behaviours from the previous four weeks on a three-point scale (1 = never, 2 = sometimes, 3 = often). The questionnaire consisted of two scales: reactive and proactive aggression. Aggressive behaviours were defined as three forms of physical aggression (i.e., kicking, hitting and pushing) and two forms of relational aggression (i.e., name calling and picking fights). To differentiate between reactive and proactive aggression, adolescents were asked to report on their motives: there were three reactive motives (i.e., “I was mad”, “I was bullied”, or “I struck back”) and three proactive motives (i.e., “I wanted to be mean”, “I took pleasure out of it”, or “I wanted to be the boss”). Total scores were calculated per scale. The internal consistencies of the scales were sufficient, ranging from 0.67 to 0.92 (see Supplementary Appendix for Cronbach’s alphas – Table S1). The Supplementary Appendix includes detailed information on the construct validity of the IRPA: a confirmatory factor analysis indicating that reactive and proactive aggression should also be treated as distinct scales in this study, and a summary of differential correlations found in previous studies for reactive and proactive aggression as measured by the IRPA with theoretically important variables.

*Brief Shame and Guilt Questionnaire* (BSGQ; Novin, & Rieffe, 2015: see Broekhof et al., 2020 for validation in DHH population): Adolescents were asked to imagine themselves occupying a described scenario, and asked to rate how ashamed or guilty they would feel on a three-point scale (1 = not at all, 2 = a little, 3 = a lot). The questionnaire consisted of 12 shame and guilt-eliciting vignettes. In six vignettes, participants were asked to indicate how ashamed they would feel, and in the other six, they were asked how guilty they would feel (e.g., Shame: “You get a very bad grade in school”; Guilt: “There is one biscuit left in the biscuit tin. You quickly put it in your mouth. Now your friend does not have a biscuit”). Total scores were calculated per scale. The internal consistencies of the scales were sufficient, ranging from 0.68 to 0.81 (see Supplementary Appendix for Cronbach’s alphas – Table S1). The Supplementary Appendix includes detailed information on the construct validity of the BSGQ: a confirmatory factor analysis indicating that shame and guilt should also be treated as distinct scales in this study, and a summary of differential correlations found in previous studies for shame and guilt as measured by the BSGQ with theoretically important variables.

## Procedure

We administered self-report questionnaires to participants at all three time points with intervals of approximately 9 months (Interval T2-T1: *M* = 9.34 months; *SD* = 0.91; Interval T3-T2: *M* = 9.87 months; *SD* = 1.15). Questionnaires were administered individually in a quiet room at the participant’s school or home. Participants were seated in front of a computer, and questions were presented one by one. For adolescents with hearing loss, all instructions and questions were accompanied by a video providing a translation in Dutch Sign Language.

The parents signed an informed consent form and an informed consent was also obtained from children who had reached the age of 12. We emphasized to both parents and children that their participation was voluntary and that we would treat all answers confidentially and anonymously. The Psychology Research Ethics Committee of Leiden University granted permission for this study.

## Statistical Analyses

To compare levels and development of proactive and reactive aggression, shame and guilt between adolescents with and without hearing loss, we used Linear Mixed Models to deal with the nested structure of our data (i.e., within-child measures). This analytic technique is also appropriate for datasets with missing data (Singer & Willett, 2003). Information about missing data in this study is reported in the Supplementary Appendix (Table S2). First, we assessed general group differences, the development of our study variables over time, and whether these developmental trajectories differed between adolescents with and without hearing loss. Using a formal modelling procedure, we fitted an unconditional means model with a fixed and random intercept. In the next step, we added group (i.e., 0 = without hearing loss, 1 = with hearing loss). In addition, we added age (centered around 9.08 years, youngest participant of the current sample) and examined three models of change: linear, quadratic, and cubic models, respectively. We added a random slope effect for the best age model, but this did not improve model fit for any model. In the last step, we added interaction with group to assess differences between groups in developmental trajectories. Preferred models had lower Akaike Information Criterion (AIC) and Bayesian Information Criterion (BIC) values. To compare whether AIC and BIC values of a subsequent model were significantly lower, the AIC and BIC values of this model were compared to the values of the model of the previous step (i.e., nested models differing one degree of freedom) using a log likelihood ratio test.

Second, linear mixed models were used to assess whether shame and guilt contributed to the linear development of reactive and proactive aggression. In the first step, we used baseline levels and change levels. Baseline levels represent the score of the participant at the first time point. (i.e., score at T1). Change scores represent the difference relative to the baseline, meaning that scores of the second time point and third time point are subtracted by the score of the first time point (i.e., T1-T1, T2-T1, and T3-T1). We also included age, group and gender (0 = boy, 1 = girl) in the analyses. This resulted in the following models:

- *Reactive aggression ~ Age + Group + Gender + Reactive aggression (baseline & change) + Shame (baseline & change) + Guilt (baseline & change).*
- *Proactive aggression ~ Age + Group + Gender + Proactive aggression (baseline & change) + Shame (baseline & change) + Guilt (baseline & change).*

In the second step, interactions with group were added (i.e., group * baseline shame; group * change shame). Again, we made a comparison between nested models by comparing AIC and BIC values (i.e., significant lower values indicate better fit). All analyses were performed in SPSS version 24.0. Graphs were made in R version 3.4.3 using the Ggplot2 function.

## Results

### Intraclass Correlations

Intraclass correlations (ICC) were calculated to test nesting of observations within individuals across the three time points. We used a two-way mixed effects model with a measure of absolute agreement and interpreted average measures. ICC were good with values of 0.76 for reactive aggression, 0.73 for proactive aggression, 0.77 for shame, and 0.79 for guilt. Pearson correlations between the averages of all study variables (i.e., of T1, T2, T3) are displayed in the Supplementary Appendix (Table S3).

## Developmental Trajectories and Group Differences

The outcomes for the best fitting model of the multilevel analyses are displayed in Table 2 (see Supplementary Appendix Table S4 for an overview of all fitted models). Individual variation is observed in the intercepts of reactive aggression, proactive aggression, shame, and guilt (see Fig. 1 of the Supplementary Appendix).

**Table 2:** Linear mixed models examining group differences and the developmental trajectory of shame, guilt, proactive aggression, and reactive aggression

Best fitting model	AIC/BIC	Intercept (se)	Group (se)	Age linear (se)	Age quadratic (se)	Group x Age (se)
Reactive aggression	4759/4769	20.92 (0.51)^***^	0.97 (0.56)	-0.38 (0.13)^**^	-	-
Proactive aggression	3971/3981	16.77 (0.30)^***^	1.47 (0.33)^***^	-0.23 (0.08)^**^	-	-
Shame	3741/3751	12.28 (0.40)^***^	-0.93 (0.31)^**^	1.25 (0.22)^***^	-0.16 (0.03)^***^	-
Guilt	3558/3568	13.15 (0.36)^***^	-2.53 (0.56)^***^	0.80 (0.20)^***^	-0.12 (0.03)^***^	0.36 (0.14)^*^

*AIC* Akaike information criterion, *BIC* Bayesian Information Criterion. Group: 0 = hearing, 1 = hearing loss

^*^p < 0.05; ^**^ *p* < 0.01; ^***^ *p* < 0.001

**Fig. 1:**
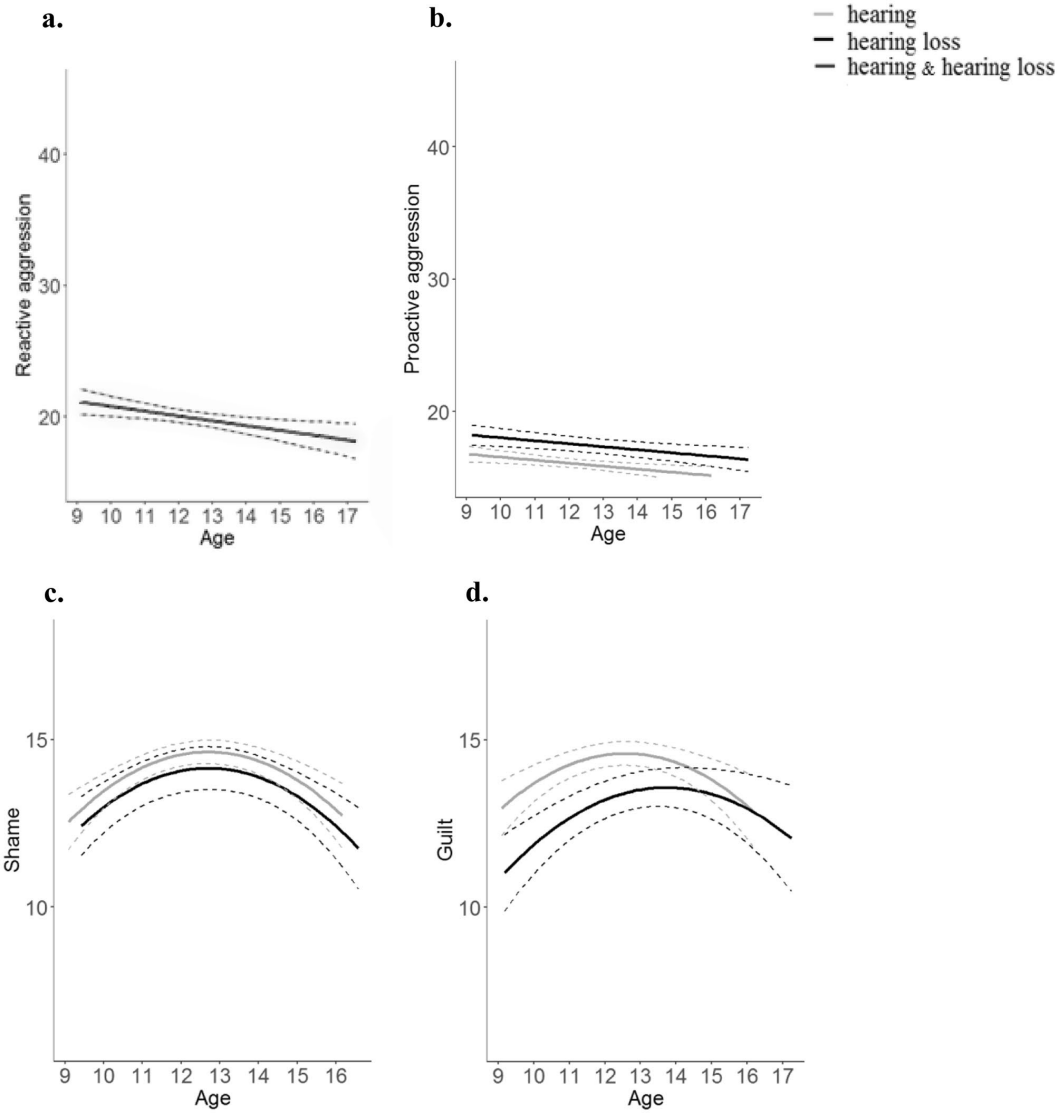
Longitudinal graphic representation of the predicted values based on the optimal fitting model for **a.** reactive aggression, **b**. proactive aggression, **c**. shame, and **d.** guilt. Lines for hearing adolescents are displayed in grey and lines for adolescents with hearing loss are presented in black. Dotted lines represent 95% confidence interval. *Note.* Figure 1a displays one line in dark grey. This indicates that lines for adolescents with and without hearing loss overlap and both groups are presented by one single line

Reactive aggression and proactive aggression were both best explained by a negative linear age-model, indicating that both types of aggression decreased over time (see Fig. 1a, b). We found no group differences for reactive aggression (*b* = 0.97, *p* = 0.084), but adolescents with hearing loss displayed higher levels of proactive aggression (*b* = 1.47, *p* < 0.001) compared to hearing adolescents (see Table 2).

The developmental trajectories of shame and guilt were best explained by a quadratic age-model. As can be seen in Fig. 1c and d, this suggests that shame and guilt peak in early adolescence. Moreover, for guilt, the optimal fitting model also included an age (quadratic) x group interaction, indicating that guilt peaks later in adolescents with hearing loss compared to adolescents without hearing loss (see Fig. 1d). As expected, adolescents with hearing loss reported lower levels of shame (*b* = -0.93, *p* < 0.001) and lower levels of guilt (*b* = -2.53, *p* < 0.001; see Table 2).

## Risk and Protective Factors for the Development of Reactive and Proactive Aggression

Linear mixed models were used to examine the predictive value of shame and guilt for the linear development of aggression. For both reactive and proactive aggression, the model without interactions fitted the data best.

As shown in Table 3, the change level for shame contributed to an increase in reactive aggression, controlling for proactive aggression. So, an increase in shame relative to T1 was associated with an increase in reactive aggression. In addition, the baseline level of shame also marginally contributed to an increase in reactive aggression (*p* = 0.058).

**Table 3:** Results of the linear mixed model on the effect of shame and guilt on aggression

	Reactive aggression		Proactive aggression	
Fixed effects				
Intercept	6.70^***^		14.82^***^	
Age	-0.22		-0.11	
Group	0.01		0.09^**^	
Gender	-0.60		0.93	
	Baseline	Change	Baseline	Change
Reactive aggression	-	**-**	0.25^***^	0.24^***^
Proactive aggression	0.71^***^	0.63^***^	-	-
Shame	0.17^#^	0.18^*^	-0.01	-0.03
Guilt	0.01	0.04	-0.24^***^	-0.18^***^
Random effects				
ID	10.98		4.35	
AIC/BIC	4572.78/4582.12		3785.80/3795.14	
df	12		12	

Adding group interactions with shame and guilt did not improve both models

^*^ *p≤*0.05; ^**^ *p≤*0.01; ^***^ *p≤*0.001; gender: 0=boys, 1=girls. ^#^ *p*=0.058

For proactive aggression, the baseline level and change in guilt contributed to a decrease in proactive aggression, controlling for reactive aggression (see Table 3). So, higher levels of guilt and an increase in guilt relative to T1 were associated with a decrease in proactive aggression.

## Discussion

Adolescence is an important transition phase from childhood to adulthood, marked by increasing responsibility to regulate one’s own behaviour and growth in social awareness (for reviews see Blakemore, 2008; Farley & Kim-Spoon, 2014). Externalizing behaviours peak during adolescence and self-evaluate emotions such as shame and guilt become part of everyday social exchange (Lansford, 2018; Petersen et al., 2015; Zeman et al., 2006). However, few studies have examined how shame and guilt contribute to the development of adolescent aggression using a longitudinal approach (Barker et al., 2006; Roos et al., 2014). In the current three-wave longitudinal study we tested: 1) the levels and development of reactive aggression, proactive aggression, shame, and guilt in adolescents with and without hearing loss, and 2) the longitudinal contribution of shame and guilt to the development of both types of aggression. We compared adolescents with and without hearing loss, and used group differences as a proxy to examine the role of social access in these interrelations.

The present study yielded several main findings, which are discussed below. In line with previous studies, reactive and proactive aggression declined throughout adolescence (Barker et al., 2006; Bongers et al., 2004; Vierikko et al., 2006). When examining how levels of self-reported shame and guilt contributed to this linear development, we found that higher levels of shame were related to increasing levels of reactive aggression over time, whereas increasing levels of guilt were related to decreasing levels of proactive aggression. These outcomes highlight the importance of differentiating between specific types of aggression in relation to shame and guilt. The developmental trend of aggression and the longitudinal associations of shame and guilt with aggression applied to both adolescents with and without hearing loss. However, adolescents with hearing loss reported higher levels of proactive aggression and lower levels of shame and guilt compared to adolescents without hearing loss. In addition, although shame and guilt peaked in early adolescence in both groups, guilt peaked later in adolescents with hearing loss compared to their peers without hearing loss.

## Risk and Protective Factors in the Development of Aggression

Importantly, our study suggests that shame is uniquely associated with higher levels of reactive aggression, and guilt is uniquely associated with lower levels of proactive aggression. Moreover, a decrease in shame contributed to a decrease in reactive aggression, whereas an increase in guilt contributed to a decrease in aggression over time. These findings support the need for longitudinal research, as changes in shame and guilt contribute to changes in aggression over time. In addition, these findings suggest it is important to differentiate between reactive and proactive aggression in relation to shame and guilt. Possibly due to the distinction between these two types of aggression, we were able to show that shame and guilt are influential in the development of these specific types of aggression with a longitudinal design. A previous longitudinal study used a general score of aggression, not differentiating between reactive and proactive aggression (Roos et al., 2014). This could have masked the unique longitudinal associations between shame and with reactive and proactive aggression specifically.

Our finding that adolescents with higher levels of shame reported increasing levels of reactive aggression adds to previous cross-sectional studies. The main theory about the path from shame to aggression posits that exposing adolescents to a shameful event initiates fury, paving the way for aggressive behaviours (Thomaes et al., 2011). Ashamed individuals are in a highly aroused state, either experiencing elevated levels of social pain or anger, hence shame’s link to reactive aggression (Lewis, 1971).

It was unexpected that shame played no discouraging role in the development of proactive aggression (Olthof, 2012). This might be caused by conceptual overlap, i.e., the shared variance of shame and guilt. Correlations to test this hypothesis indicate that shame correlated with proactive aggression when guilt was not included in the analysis to parse out this shared variance (see Table S3 of the Supplementary Appendix). This suggests that shame is only negatively associated with lower levels of proactive aggression when guilt is not accounted for. Additionally, proactive aggression is calculated behaviour and concerns intentionally violating others. Proactive aggression is therefore linked to a lack of morality (Arsenio et al., 2009). It is possible that we did not found a link between shame and proactive aggression because it is often viewed as less of a moral emotion relative to guilt. In contrast to guilt, shame does not only occur in situations in which moral standards are violated but also in non-moral situations in which one feels devalued by others (Tangney & Dearing, 2002).This study adds to literature explaining the developmental trajectory of proactive aggression by mapping the role of guilt in this development. In our study, more guilt (higher initial and increasing levels) contributed to a decrease in proactive aggression. This finding is in line with cross-sectional studies and suggests that adolescents with higher levels of guilt are less inclined to behave aggressively without being provoked, because of the negative emotional consequences of aggressive behavior for themselves. As expected, there were no longitudinal associations between guilt and reactive aggression. There are several possible explanations why guilt attributions are not related to the development of reactive aggression. Previous research has indicated that emotionally aroused individuals are more likely to act impulsively, reflected by a preference for instant small gratification, even in the face of delayed negative consequences (Sohn et al., 2015; Peters et al., 2006). Thus, if one feels provoked by someone, it is more tempting to retaliate, even if one anticipates consequential guilt. At the same time, from middle childhood onwards, individuals judge aggression to defend oneself (i.e., reactive aggression) as more morally justifiable than aggression to obtain selfish instrumental goals (i.e., proactive aggression; Jambon & Smetana, 2014). Anticipating the consequences of engagement in reactive aggression would therefore result in less intense guilt attributions, as compared to engagement in proactive aggression, minimizing the protective influence of guilt for reactive aggression.

The unique associations of shame with reactive aggression and guilt with proactive aggression were similar in adolescents with and without hearing loss. Thus, the level of social access does not seem to alter the role of shame and guilt on the development of aggression. Can lower levels of shame and guilt therefore explain the higher incidence of proactive aggression in adolescents with hearing loss? Similar to the hearing group, lower levels of guilt were linked to the development of higher levels of proactive aggression in adolescents with hearing loss. Given that levels of guilt were lower for children with hearing loss, it is not surprising that these adolescents were indeed found to have a higher level of proactive aggression. In contrast, we found that higher levels of shame are related to higher levels of reactive aggression. With lower levels of shame, compared to their hearing peers, adolescents with hearing loss do not seem to be at risk for the development of reactive aggression. However, more research is needed to determine the effect of social access on the longitudinal interrelations between shame, guilt and reactive and proactive aggression. One recommendation for future studies is to focus on the development of shame and guilt in younger children of about three to four years old, in a developmental stage in which shame and guilt are still developing and aggression is also quite common (Girard et al., 2019; Teymoori et al., 2018).

## Developmental Patterns of Shame and Guilt

Shame and guilt peak in early adolescence: the reported intensity of both shame and guilt increase from preadolescence to early adolescence and decrease thereafter into middle adolescence. This quadratic pattern is compatible with studies showing that peer sensitivity is highest around early adolescence (e.g., Steinberg, 2008). Fear of peer rejection, or a strong desire to belong to a peer group, could foster perspective taking abilities and the willingness to behave in accordance with social norms and values (van Hoorn et al., 2016; Newman et al., 2007). Early adolescents seem particularly reluctant to harm another peer or to behave incompetently in the presence of others, indicating higher levels of shame and guilt in this adolescent phase (Reimer, 1996).

Adolescents with hearing loss reported lower levels of shame and guilt in general, and a more protracted development of guilt, compared to adolescents without hearing loss. This finding highlights the need for social learning. In order for shame and guilt to arise, there must be an appreciation for the perspectives and feelings of others and an appreciation for social rules and standards (Tangney & Dearing, 2002). Children and adolescents with hearing loss are consistently found to be less aware of others’ perspectives and feelings, due to less access to the social world (Jones et al., 2015; Ketelaar et al., 2015). Consequently, it is possible that adolescents with hearing loss do not foresee the negative evaluations of others, or any negative emotional consequences for others as a result of their aggressive behaviour, making it less likely that shame and guilt will occur.

It remains speculative why the developmental pace of guilt peaks later in adolescents with hearing loss, whereas the developmental pace of shame is in line with adolescents without hearing loss. An explanation may lie in the differences between shame and guilt. Whereas shame is focused on oneself in light of a negative evaluation by others, guilt is focused on the other, thus requiring stronger perspective taking capacity. It could be that the switch from perspective taking with a focus on the self to perspective taking with a focus on the other is more challenging for adolescents with less access to the social world. Future studies need to unravel whether adolescents with less access to the social world could benefit from training in perspective taking abilities, in order to prevent lower levels and a slower developmental pace of guilt.

## Limitations and Strengths

The present study has several strengths, but there are also some limitations that need to be addressed. First, the levels of aggression were generally low in our adolescent sample, as is frequently observed in studies with non-clinical samples (see Fig. 1a and b; Barker et al., 2006; Roos et al., 2014). Nevertheless, there was sufficient intra- and inter-individual change to map developmental changes in aggression, and to examine the contribution of shame and guilt to these changes in aggression. Second, this study relied solely on self-report measures, increasing the risk for common-method variance bias (Podsakoff et al., 2003). Future studies should use varying measurement methods and sources by also including observational measures or peer reports. Third, we have asked adolescents to rate how ashamed or guilty they would feel if they transgressed a social norm (i.e., damaging a classmate or not knowing what to say in front of a group of people). Thus, we did not measure shame and guilt in reaction to an aggressive act. This might have influenced our results, although we can only speculate whether the measurement method had an effect on the outcomes regarding the links of shame and guilt with aggression. If we compare cross-sectional studies that have measured shame and guilt in response to the broader category of norm-violating behavior (e.g., Roberts et al., 2014; Roos et al., 2015; Furukawa et al., 2012) with studies that have used shame and guilt following aggressive behavior (e.g., Olthof, 2012; Thomaes et al., 2008) the results regarding the link with aggression are comparable. However, a meta-analysis is needed to unravel the potential influence of this measurement method on our results.

Among the strengths of this study is the longitudinal design, with three measurements in early adolescence and approximately 9 months in between. It enabled us to map developmental changes in aggression, and to examine the longitudinal contribution of shame and guilt to these changes. Another strength of this study is that we included a sample of adolescents with hearing loss as a proxy for access to the social world. This unique approach made it possible to examine the role of social access on the development of aggression and, shame and guilt.

## Conclusion

The current longitudinal study showed that adolescents with and without hearing loss engage in less reactive and proactive aggression as they mature from early to middle adolescence. However, reported levels of proactive aggression are elevated in adolescents with hearing loss. In addition, shame and guilt peaked in early adolescents but adolescents with hearing loss reported lower levels of these social emotions compared to hearing peers. These group differences emphasize the important role of access to the social world in the development of shame and guilt.

Our study suggests that shame is an important risk factor in the development of reactive aggression, whereas guilt is an important protective factor in the development of proactive aggression for both adolescents with and without hearing loss. Future studies should determine whether promoting perspective taking with the focus on others, as is characteristic for guilt, as opposed to perspective taking with the focus on the self as is characteristic for shame, could provide means for developing interventions that successfully prevent aggressive behaviour in adolescence.

## Supplementary Information

Below is the link to the electronic supplementary material.

Supplementary file1 (DOCX 500 KB)
