# Supplementary material for: The Roles of Shame and Guilt in the Development of Aggression in Adolescents With and Without Hearing Loss
Source: Res Child Adolesc Psychopathol. 2021 Feb 24;49(7):891–904. doi: 10.1007/s10802-021-00769-1 (PMC8154774; doi:10.1007/s10802-021-00769-1)
Supplement: Supplementary file 1 — Supplementary file1 (DOCX 500 KB) [file 10802_2021_769_MOESM1_ESM.docx]

**Supplementary Appendix.**

***A detailed description on the construct validity of the IRPA and BSGQ.***

**Structural validity**

Confirmatory factor analyses were conducted to examine whether reactive and proactive aggression and whether shame and guilt should be treated as distinct scales in our study as previous studies suggest (e.g., Cima et al., 2013; Kempes et al., 2005; Olthof, 2012). These analyses were conducted in R version 3.6.1, using packages lavaan and semTools.

We first examined the fit to a one-factor model and compared it to a two-factor model (i.e., distinct scales for reactive/proactive aggression and shame/guilt). Model fit was assessed using the χ^2^ /df ratio, the comparative fit index (CFI), the Tucker– Lewis Index (TLI) and the root mean square error of approximation (RMSEA). A χ^2^ /df ratio of 3 or lower indicates good fit. CFI and TLI values above .90 indicate acceptable fit and values above .95 represent good fit. For RMSEA, values below .08 indicate reasonable fit and values below .05 represent good fit.

***Instrument for Reactive and Proactive Aggression (IRPA):*** To conduct confirmatory factor analyses to evaluate the factor structure of the IRPA questionnaire six parcels scores were created, which are mean scores per motive for aggression (e.g., “I was mad”, “I wanted to be mean”). In a first step, a one-factor model including all aggression items was analysed and fit indices showed inadequate fit, *χ^2^*(*df* = 9, *N* = 307) = 158.20, *χ*^2^/*df* = 17.58, CFI = .79, TLI = .64 and RMSEA = .23. In a second step, the assumed two-factor model (with items for reactive and proactive aggression separately) was tested and demonstrated a good fit, *χ*^2^(*df* = 8, *N* = 307) = 17.50, χ^2^/df = 2.19, CFI = .98, TLI = .97 and RMSEA = .06. Based on these fit measures, the two-factor model should be preferred over the one-factor model (Δ*χ*^2^(1) = 140.70, *p* < .001).

***Brief Shame and Guilt Questionnaire:*** In a first step, a one-factor model including all 12 shame and guilt items was analysed and fit indices ranged from acceptable to inadequate fit, *χ*^2^(df = 54, *N* = 305) = 203.29, *χ^2^/df* = 3.76, CFI = .92, TLI = .90 and RMSEA = .10. In a second step, the assumed two-factor model (with 6 items for shame and 6 items for guilt separately) was tested and demonstrated a good fit, *χ*^2^(*df* = 53, *N* = 305) = 133.97, *χ^2^/df* = 2.53, CFI = .96, TLI = .95 and RMSEA = .07. Based on these fit measures, the two-factor model should be preferred in comparison to the one-factor model (Δ*χ*^2^(1) = 69.32, *p* < .001).

These analyses indicate that reactive and proactive aggression, and shame and guilt should be treated as distinct scales in our study.

**Convergent and discriminant validity based on previous studies**

**IRPA**. A previous validation study has indicated that the IRPA is a reliable and valid instrument to use with children of nine years and older (Rieffe et al., 2016). Rieffe and colleagues (2016) found differential correlations with theoretically important variables for the scales reactive and proactive aggression. Reactive aggression was uniquely related to lower levels of self-reported emotion awareness and self-esteem. Proactive aggression was uniquely associated with a higher levels of conduct problems as reported by parents, and antisocial behaviours as reported by peers. Another study using the IRPA found a positive correlation for anger dysregulation with reactive aggression, but not with proactive aggression in adolescents with and without a developmental language disorder (van den Bedem, Dockrell, van Alphen & Rieffe, 2020).

**BSGQ.** Two separate studies confirmed that the BSGQ can used validly in both adolescents with and without hearing loss (Novin & Rieffe, 2015; Broekhof et al., 2020). Novin and Rieffe (2015) validated the use of the BSGQ in a hearing sample of 9 to 14-year olds. In their study, shame was uniquely associated with higher levels of social anxiety and worry, and guilt was uniquely associated with lower levels of conduct problems as reported by children and teachers. Broekhof and colleagues (2020) validated the use of the BSGQ in 9 to 15-year olds with hearing loss. This study indicated that shame was uniquely associated with more a lower self-esteem, and guilt was uniquely associated with higher levels of delinquency and psychopathic behaviours.

Based on previous studies, we can conclude that both types of aggression and shame and guilt as measured by the IRPA and BSGQ are differently related to theoretically important variables in adolescence. This indicates that both the IRPA and BSGQ display convergent and discriminant validity.

**References:**

Broekhof, E., Kouwenberg, M., Oosterveld, P., Frijns, J. H. M., & Rieffe, C. (2020). Use of the Brief Shame and Guilt Questionnaire in deaf and hard of hearing children and adolescents. Assessment, 24, 194-205. doi: 10.1177/1073191117725169

Novin, S., & Rieffe, C. (2015). Validation of the Brief Shame and Guilt Questionnaire for Children. Personality and Individual Differences, 85, 56-59. doi:10.1016/j.paid.2015.04.028

van den Bedem, N. P., Dockrell, J. E., Van Alphen, P. M., & Rieffe, C. (2020). Emotional competence mediates the relationship between communication problems and reactive externalizing problems in children with and without Developmental Language Disorder: A longitudinal study. International Journal of Environmental Research and Public Health, *17*, 6008. doi: 10.3390/ijerph17166008

**
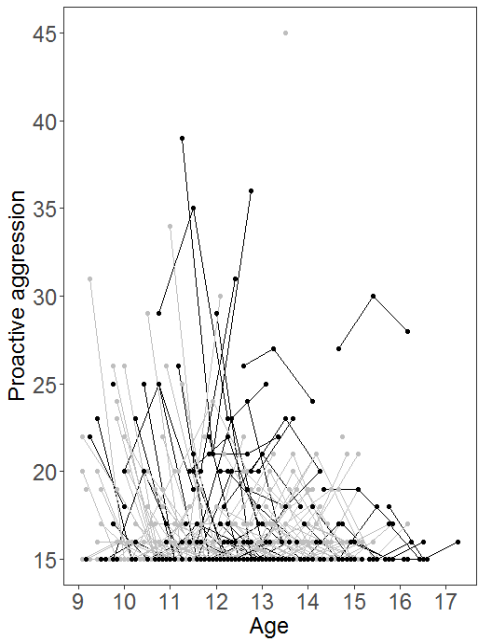

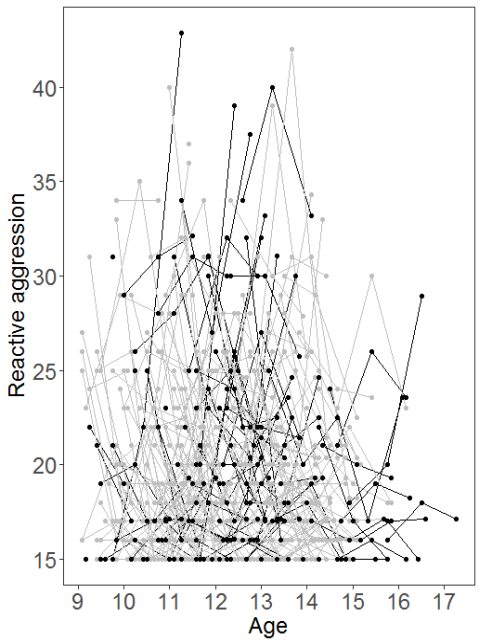
A. B.**

**
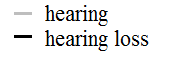
**

**
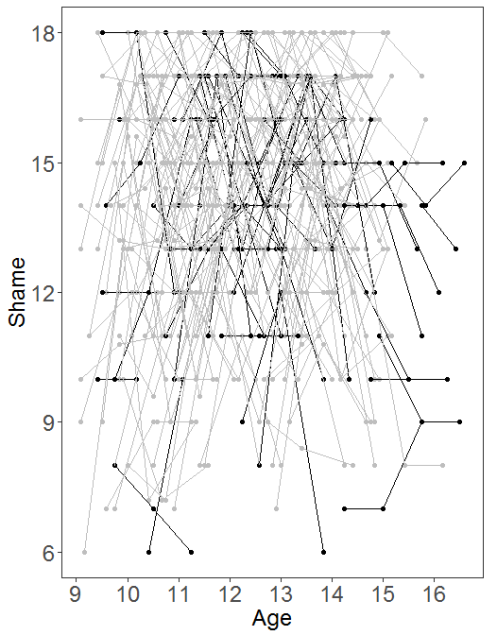

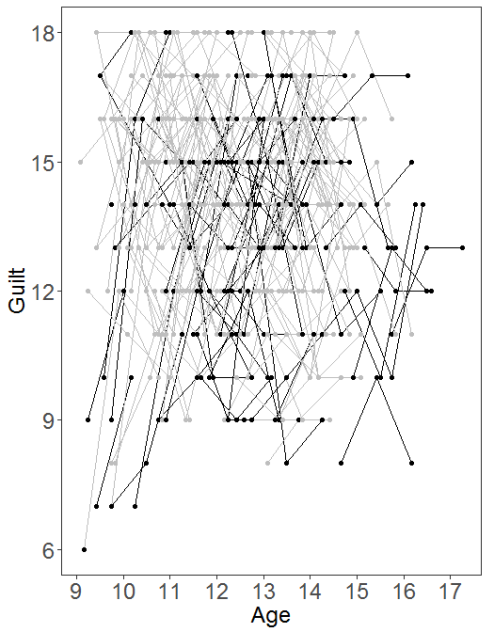
C. D.**

**Figure 1.** Longitudinal graphic representation of age at the three time points of reactive aggression, proactive aggression, shame and guilt. Each participant is presented by an individual line and each time point is presented by a point. Adolescents with hearing loss are displayed in black and hearing adolescents in grey. **1A.** reactive aggression. **1B.** proactive aggression. **1C.** shame. **1D.** guilt.

S1. *Psychometric properties and mean scores of reactive aggression, proactive aggression, shame and guilt at the three time points per group.*

|  | No. of items | Range | Cronbach’s *α* | | Mean scores (*SD*) | |
| --- | --- | --- | --- | --- | --- | --- |
|  |  |  | HL | Hearing | HL | Hearing |
| **Time 1** |  |  |  |  |  |  |
| Reactive aggression | 15 | 15-45 | .89 | .89 | 20.36 (5.28) | 20.41 (5.14) |
| Proactive aggression | 15 | 15-45 | .87 | .90 | 18.23 (4.50) | 16.47 (3.39) |
| Shame | 6 | 6-18 | .81 | .78 | 13.00 (3.33) | 14.03 (2.92) |
| Guilt | 6 | 6-18 | .80 | .69 | 12.55 (3.08) | 14.07 (2.50) |
|  |  |  |  |  |  |  |
| **Time 2** |  |  |  |  |  |  |
| Reactive aggression | 15 | 15-45 | .91 | .90 | 20.17 (5.52) | 19.40 (4.90) |
| Proactive aggression | 15 | 15-45 | .92 | .67 | 16.95 (4.00) | 15.55 (1.28) |
| Shame | 6 | 6-18 | .69 | .68 | 13.86 (2.58) | 14.61 (2.61) |
| Guilt | 6 | 6-18 | .78 | .69 | 12.96 (2.72) | 14.38 (2.40) |
|  |  |  |  |  |  |  |
| **Time 3** |  |  |  |  |  |  |
| Reactive aggression | 15 | 15-45 | .92 | .87 | 21.09 (6.31) | 18.68 (4.21) |
| Proactive aggression | 15 | 15-45 | .90 | .77 | 16.94 (3.72) | 15.75 (1.80) |
| Shame | 6 | 6-18 | .68 | .75 | 12.70 (2.72) | 14.12 (2.69) |
| Guilt | 6 | 6-18 | .69 | .68 | 13.27 (2.51) | 14.51 (2.51) |

*Abbreviations*: HL = Hearing loss; SD: Standard deviation.

S2. *An overview of missing data.*

|  | Participants | | Missing | |  |  |
| --- | --- | --- | --- | --- | --- | --- |
|  | HL | Hearing | HL  Count | HL  Percentage | Hearing  Count | Hearing  Percentage |
| Time 1 | *n =* 80 | *n =* 227 |  |  |  |  |
| Age | 80 | 227 | 0 | 0.00% | 0 | 0.00% |
| Reactive aggression | 78 | 227 | 2 | 2.50% | 0 | 0.00% |
| Proactive aggression | 78 | 227 | 2 | 2.50% | 0 | 0.00% |
| Shame | 78 | 227 | 2 | 2.50% | 0 | 0.00% |
| Guilt | 78 | 227 | 2 | 2.50% | 0 | 0.00% |
| Time 2 | *n =* 78 | *n =* 197 | 2 | 2.50% | 30 | 13.22% |
| Age | 78 | 197 | 2 | 2.50% | 30 | 13.22% |
| Reactive aggression | 78 | 197 | 2 | 2.50% | 30 | 13.22% |
| Proactive aggression | 78 | 197 | 2 | 2.50% | 30 | 13.22% |
| Shame | 74 | 194 | 4 | 5.00% | 33 | 14.54% |
| Guilt | 74 | 194 | 4 | 5.00% | 33 | 14.54% |
| Time 3 | *n =* 64 | *n =* 166 | 16 | 20.00% | 61 | 26.87% |
| Age | 64 | 166 | 16 | 20.00% | 61 | 26.87% |
| Reactive aggression | 64 | 166 | 16 | 20.00% | 61 | 26.87% |
| Proactive aggression | 64 | 166 | 16 | 20.00% | 61 | 26.87% |
| Shame | 63 | 166 | 17 | 21.25% | 61 | 26.87% |
| Guilt | 63 | 166 | 17 | 21.25% | 61 | 26.87% |

*Note.* HL = Hearing loss.

S3. *Correlations between the average score (of time1, time2, time3) of social emotions with aggression.*

|  | Proactive aggression | Shame |  | Guilt |  |
| --- | --- | --- | --- | --- | --- |
|  |  |  | Partial^a^ |  | Partial^a^ |
| Reactive aggression | .43^***^ | .01 | .10 | -.13^*^ | -.16^**^ |
| Proactive aggression |  | -.13^*^ | .04 | -.29^***^ | -.26^***^ |

*Note.* ^*^*p* < .05, ^**^ *p* < .01, ^***^ *p* <.001.

^a^ Partial correlations were corrected for either shame or guilt.

S4. *Linear mixed models examining the developmental trajectory of reactive aggression, proactive aggression, shame and guilt.*

|  | Reactive aggression | Proactive aggression | Shame | Guilt |
| --- | --- | --- | --- | --- |
| **Model 1** |  |  |  |  |
| Intercept | 19.91^***^ | 16.40^***^ | 13.97^***^ | 13.90^***^ |
| AIC/  BIC | 4768.54/  4777.93 | 3993.65/  4003.04 | 3773.36/3782.74 | 3592.39/3601.76 |
| Df | 3 | 3 | 3 | 3 |
|  |  |  |  |  |
| **Model 2** |  |  |  |  |
| Intercept | 19.69^***^ | 16.03^***^ | 14.24^***^ | 14.26^***^ |
| Group | .84 | 1.39^***^ | -1.00^***^ | -1.34^***^ |
| AIC/  BIC | 4765.60/  4774.99 | 3976.54/  3985.93 | 3763.61/3772.98 | 3570.19/3579.56 |
| df | 4 | 4 | 4 | 4 |
|  |  |  |  |  |
| **Model 3** |  |  |  |  |
| Intercept | **20.92^***^** | **16.77^***^** | 13.79^***^ | 13.89^***^ |
| Group | **.97** | **1.47^***^** | -1.05^***^ | -1.38^***^ |
| Age(linear) | **-.38^**^** | **-.23^**^** | .14 | .12 |
| AIC/  BIC | **4759.18/**  **4768.57** | **3971.40/**  **3980.78** | 3763.37/3772.72 | 3570.54/3579.91 |
| df | **5** | **5** | 5 | 5 |
|  |  |  |  |  |
| **Model 4** |  |  |  |  |
| Intercept | 20.70^***^ | 17.56^***^ | **12.28^***^** | 12.95^***^ |
| Group | .99 | 1.41^***^ | **-.93**^**^ | -1.31^***^ |
| Age(linear) | -.22 | -.81^***^ | **1.25^***^** | .80^***^ |
| Age(quadratic) | -.02 | .08^*^ | **-.16^***^** | -.10^***^ |
| AIC/  BIC | 4762.90/  4772.28 | 3970.55/  3979.94 | **3741.23/3750.60** | 3562.70/3572.07 |
| df | 6 | 6 | **6** | 6 |
|  |  |  |  |  |
| **Model 5** |  |  |  |  |
| Intercept | 21.07^***^ | 18.28^***^ | 12.13^***^ | 12.50^***^ |
| Group | 1.02 | .15^***^ | -.94^**^ | -1.34^***^ |
| Age(linear) | -.76 | -1.84^***^ | 1.47^**^ | 1.46^***^ |
| Age(quadratic) | .16 | .44^**^ | -.24 | -.33^*^ |
| Age(cubic) | -.02 | -.03^*^ | .01 | .02 |
| AIC/  BIC | 4767.95/  4777.34 | 3972.26/  3981.65 | 3747.72/3757.08 | 3566.58/3575.93 |
| df | 7 | 7 | 7 | 7 |
|  |  |  |  |  |
| **Model 6** |  |  |  |  |
| Intercept | 21.29^***^ | 16.55^***^ | 12.32^***^ | **13.15**^***^ |
| Group | -.33 | 2.23^***^ | -1.16 | **-2.53**^***^ |
| Age (linear) | -.50^***^ | -.16 | 1.25^***^ | **.80**^***^ |
| Age(quadratic) | X | X | -.17^***^ | **-.12**^***^ |
| Age x Group | .38 | -.22 | .07 | **.36**^*^ |
| AIC/  BIC | 4758.13/  4767.52 | 3971.43/  3980.82 | 3738.91/3752.27 | **3558.42/3567.78** |
| df | 6 | 6 | 7 | **7** |

^*^*p* < .05, ^**^ *p* < .01, ^***^ *p* <.001.

Values for the best fitting model are displayed in bold.
